# Meat Consumption and Risk of Oral Cavity and Oropharynx Cancer: A Meta-Analysis of Observational Studies

**DOI:** 10.1371/journal.pone.0095048

**Published:** 2014-04-15

**Authors:** Jing Xu, Xin-xin Yang, Yun-gang Wu, Xiao-yu Li, Bo Bai

**Affiliations:** 1 Department of Gynecology, The Affiliated Hospital of Jining Medical University, Jining, Shandong Province, People’s Republic China; 2 Department of ENT, The Affiliated Hospital of Jining Medical University, Jining, Shandong Province, People’s Republic China; 3 Department of Physiology, Jining Medical University, Jining, Shandong Province, People’s Republic China; Tor Vergata University of Rome, Italy

## Abstract

**Purpose:**

High meat consumption, especially red and processed meat consumption is associated with an increased risk of several cancers, however, evidence for oral cavity and oropharynx cancer is limited. Thus, we performed this meta-analysis to determine the association between intakes of total meat, processed meat, red meat, and white meat, and the risk of oral cavity and oropharynx cancer.

**Methods:**

Electronic search of Pubmed, Embase, and Cochrane Library Central database was conducted to select relevant studies. Fixed-effect and random-effect models were used to estimate summary relative risks (RR) and the corresponding 95% confidence intervals (CIs). Potential sources of heterogeneity were detected by meta-regression. Subgroup analyses and sensitivity analysis were also performed.

**Results:**

12 case–control studies and one cohort study were included in the analyses, including 501,730 subjects and 4,104 oral cavity and oropharynx cancer cases. Pooled results indicated that high consumption of total meat, red meat, and white meat were not significantly associated with increased risk of oral cavity and oropharynx cancer (RR = 1.14, 95% CI[0.78–1.68]; RR = 1.05, 95% CI[0.66, 1.66] and RR = 0.81, 95% CI[0.54, 1.22], respectively), while the high consumption of processed meat was significantly associated with a 91% increased risk of oral cavity and oropharynx cancer (RR = 1.91, 95% CI [1.19–3.06]). Sensitivity analysis indicated that no significant variation in combined RR by excluding any of the study, confirming the stability of present results.

**Conclusions:**

The present meta-analysis suggested that high consumption of processed meat was significantly associated with an increased risk of oral cavity and oropharynx cancer, while there was no significantly association between total meat, red meat or white meat and the risk of oral cavity and oropharynx cancer. More prospective cohort studies are warranted to confirm these associations.

## Introduction

Oral cavity and oropharynx cancer are the tenth most common cancer and seventh most common cause of cancer-related mortality worldwide [1], [2]. The primary risk factors for oral cavity and oropharynx cancer have been well documented, including betel-quid chewing, tobacco smoking, and alcohol consumption [2]. However, the roles of many putative risk factors in etiology of oral cavity and oropharynx cancer remain unclear. Numerous studies have shown that diet may also be of etiologic importance. As we know, meat plays an important part in a healthy, balanced diet, and high meat consumption, especially red and processed meat consumption has been found to be associated with an increased risk of several malignancies, such as colorectal cancer [3], esophageal cancer [4], lung cancer [5], bladder cancer [6], and renal cancer [7]. The association of meat consumption as a potential risk for oral cavity and oropharynx cancer has been studied in several observational studies, however, these studies yielded different or even controversial results. For example, Lissowska J et al found that consumption of total meat, processed meat were inversely associated with the risk of oral cavity and oropharynx cancer [8], however, other investigators found that meat consumption was significantly associated with increased risk of oral cavity and oropharynx cancer [9], [10], [11]. Therefore, to better characterize the association between meat consumption and the risk of oral cavity and oropharynx cancer, we conducted a comprehensive meta-analysis of the current observational studies.

## Methods

### Literature Search

The present meta-analysis was conducted following the Preferred Reporting Items for Systematic reviews and Meta-Analyses guidelines(PRISMA) [12], and the meta-analysis of observational studies in epidemiology (MOOSE) guidelines [13]. A literature search was carried out using Pubmed, Embase, and Cochrane Library Central database between January 1966 and May 2013. There was no restriction of origin and language. Search terms included: “meat” or “lamb” or “beef” or “pork” or “bacon” or “poultry” or “chicken” and ‘‘cancer(s)’’ or ‘‘neoplasm(s)’’ or ‘‘malignancy(ies)’’ and “oral” or “mouth” or “pharynx” or “pharyngeal” or “oropharyngeal”. The reference lists of each study included in this meta-analysis and previous reviews were manually examined to identify additional relevant studies.

### Study selection

Two reviewers independently selected eligible studies. Disagreement between the two reviewers was settled by discussing with the third reviewer. Studies were selected if they met our criteria (i) had a case-control or cohort design; (ii) evaluated the association between meat (total meat, red meat, processed meat, or white meat) consumption and the risk of oral cavity and oropharynx cancer, and (iii) presented odds ratio (OR), relative risk (RR), or hazard ratio (HR) estimates with its 95% confidence interval (CI).When there were multiple publications from the same population, only data from the most recent report was included in the meta-analysis and the others were excluded. Studies reporting different measures of RR like risk ratio, rate ratio, hazard ratio, and odds ratio were included in the meta-analysis. In practice, these measures of effect yield a similar estimate of RR, since the absolute risk of oral cavity and oropharynx cancer is low.

### Data extraction and methodological quality assessment

The following data was collected by two reviewers independently using a purpose-designed form: name of the first author, publishing time, study region, study design, study period, number of cancer cases and subjects, dietary assessment method, the exposure of meat intake, quantity of intake, the study-specific adjusted ORs, RRs, or HRs with their 95% CIs for the highest category of meat consumption versus the lowest, confounding factors for matching or adjustments.

We used Newcastle-Ottawa scale to assess the methodologic quality of cohort and case-control studies. The Newcastle-Ottawa Scale contains eight items that are categorized three categories: selection (four items, one star each), comparability (one item, up to two stars), and exposure/outcome (three items, one star each). A ‘‘star’’ presents a ‘‘high-quality’’ choice of individual study. The full score was 9 stars, and the high-quality study was defined as a study with≥6 awarded stars.

### Data synthesis and analysis

Heterogeneity was assessed using the Cochran Q and I^2^ statistics. For the Q statistic, a P value<0.10 was considered statistically significant for heterogeneity; for the I^2^ statistic, heterogeneity was interpreted as absent (I^2^: 0%–25%), low (I^2^: 25.1%–50%), moderate (I^2^: 50.1%–75%), or high (I^2^: 75.1%–100%) [14]. The overall analysis including all eligible studies was performed first, and subgroup analyses were performed according to (i) Study location(South America, North America, Europe, and Asia), and (ii)number of confounding factors (n≥7, n≤6), adjustment for alcohol intake (yes, no), adjustment for BMI (yes, no), adjustment for education(yes, no), adjustment for fruit and/or vegetable intake(yes, no), to examine the impact of these factors on the associations. When substantial heterogeneity was detected, the summary estimate based on the random-effect model (DerSimonian –Laird method) [15] was reported, which assumed that the studies included in the meta-analysis had varying effect sizes. Otherwise, the summary estimate based on the fixed-effect model (the inverse variance method) [16] was reported, which assumed that the studies included in the meta-analysis had the same effect size. To test the robustness of the associations and characterize possible sources of statistical heterogeneity, sensitivity analysis was carried out by excluding studies one-by-one and analyzing the homogeneity and effect size for all of the rest studies. To better investigate the possible sources of between-study heterogeneity, a meta-regression analysis was performed [17]. An univariate model was established, and then variables with P values ≥0.1 were entered into a multivariable model. Publication bias was assessed using Begg and Mazumdar adjusted rank correlation test and the Egger regression asymmetry test [18], [19]. All analyses were performed using Stata version 11.0 (StataCorp, College Station, TX).

## Results

### Literature search and study characteristics

**Figure 1:**
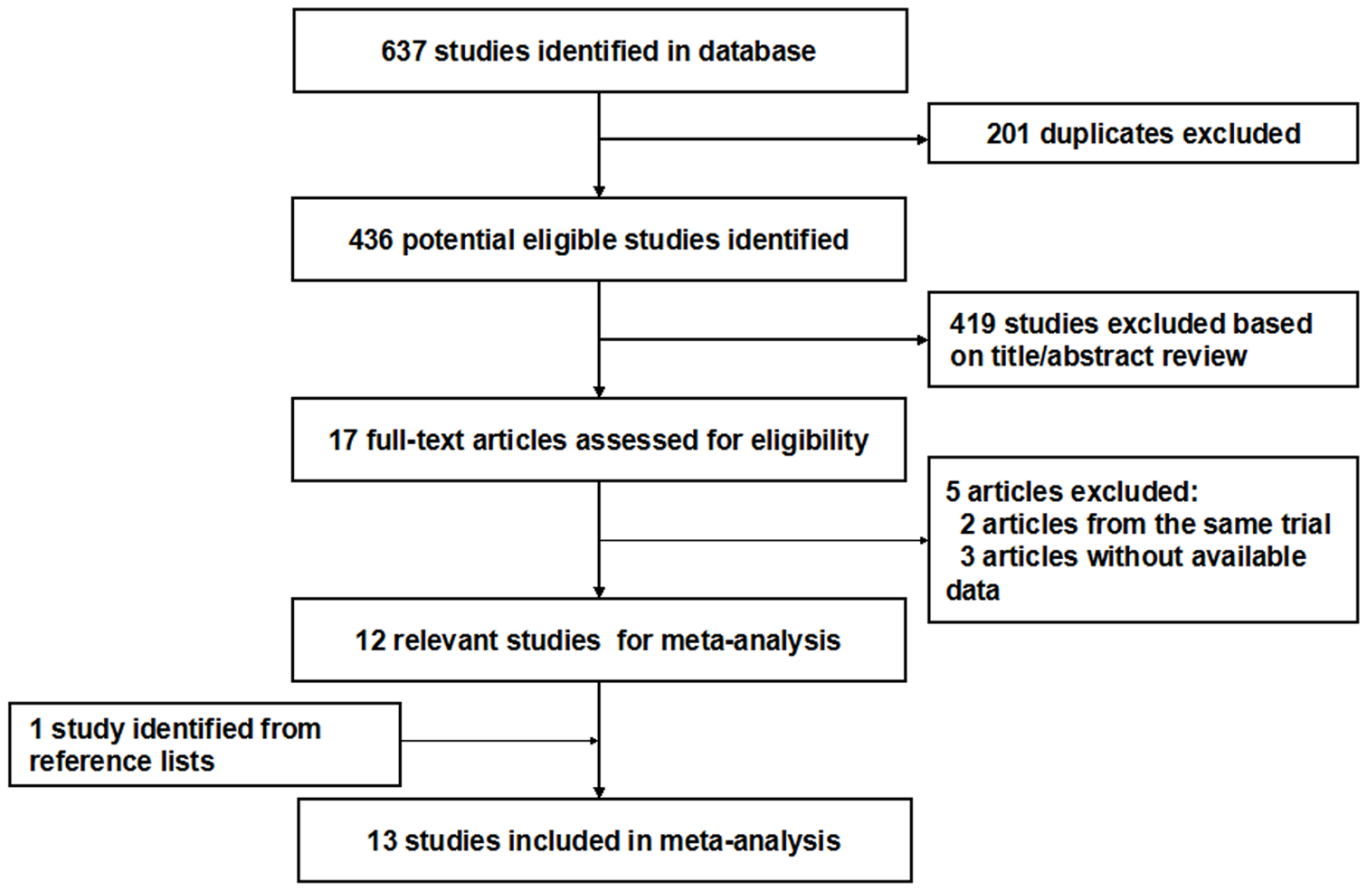
Flow diagram of screened, excluded, and analysed publications.

The detailed steps of our literature search are shown in Figure 1. The search strategy generated 637 citations. On the basis of the titles and abstracts, we identified 17 relevant articles. After further evaluation, three studies were excluded for lack of available data, and two studies were excluded because they were from the same population. One study was identified from the reference lists. At last, a total of 13 eligible studies published between 1992 and 2012 were identified, including 12 case–control studies [8], [9], [10], [11], [20], [21], [22], [23], [24], [25], [26], [27] and one cohort study [28](Baseline data and other details of included studies are shown in Table 1). A total of 501,730 subjects, including 4,104 oral cavity and oropharynx cancer cases were involved. Of the 13 included studies, six studies were conducted in Europe [8], [21], [22], [23], [24], [25], three studies in Asia [11], [26], [27], three studies in South America [9], [10], [20], and the remaining one study in North America [28]. Among the 12 case-control studies, only one study was population based [27], and the others were hospital based. Most studies used food frequency questionnaires(FFQ) for the assessment of meat consumption. All studies adjusted for smoking, and most studies adjusted for some potential confounders, including age, sex, and alcohol consumption. The NOS scores for the included studies ranged from 4 to 8; nine studies were deemed to be of a high quality (≥6) (shown in Table 1).

**Table 1:** Characteristics of observational studies of the relation between meat intakes and risk of oral cavity and oropharynx cancer included in the meta-analysis.

Author	Publication year	Country	Study design	Study period	Dietary assessments	Cases/Subjects	Type of meat	Units and comparison groups	Confounders for adjustment	NOS score
De Stefani E	2012	Uruguay	hospital-based case–control study	1996–2004	FFQ 64 items	56/940	processed meat	g/day 11.5–28.2 vs ≤11.4 ≥28.3 vs ≤11.4	age, residence, BMI, smoking status, alcohol drinking, mate consumption, total energy, total vegetables and fruits, total white meat, and red meat intakes.	7
Daniel CR	2011	USA	cohort study	1995–1996	FFQ 124 items	1,305/492,186	Poultry	Q5 vs Q1	age, sex, education, marital status, family history of cancer, race, BMI, smoking status, frequency of vigorous physical activity, menopausal hormone therapy in women, intake of alcohol, fruit, vegetables, fish, red meat, and total energy	8
Sapkota A	2008	central and eastern Europe	hospital-based case–control study	1999–2003	FFQ 23 items	378/1,606	Poultry, red meats	Tertile 3 vs Tertile 1	age, country, gender, tobacco pack-years, education, BMI, frequency of alcohol consumption, tertiles of total vegetable consumption, and tertiles of total fruit consumption	7
Levi F	2004	Switzerland	hospital-based case–control study	1992–2002	FFQ 79 items	316/1,587	Processed meat	Frequency/week 0.8–1.5 vs <0.8 1.6–3.2 vs <0.8 >3.2 vs <0.8	age, sex, education, tobacco smoking, alcohol drinking, total energy intake, fruit and vegetable intake,BMI, and physical activity .	7
Toporcov TN	2004	Brazil	hospital-based case–control study	2003	FFQ 41 items	70/140	pepperoni,bacon, red meat	high vs low	sex, age, smoking status, frequency for the use of dental prosthesis	6
Lissowska J	2003	Poland	hospital-based case–control study	1997–2000	FFQ 25 items	122/246	Total meat, processed meat	Tertile 3 vs Tertile 1	gender, age, residence, drinking and smoking habits	6
Rajkumar T	2003	India	hospital-based case–control study	1996–1999	FFQ 21 items	591/1,773	Total meat, processed meat	Servings/week 1-2 vs <1 >2 vs <1	sex, age, centre, education, chewing, smoking and drinking habits	6
Sánchez MJ	2003	Spain	hospital-based case–control study	1996–1999	FFQ 25 items	375/750	Total meat, processed meat	Servings/week 2-5 vs <1 6 vs <1	gender, age, centre, years of schooling, smoking and drinking habits	6
Escribano Uzcudun A	2002	Spain	hospital-based case–control study	1990–1995	interview-administered questionnaire	232/464	Total meat	high vs low	tobacco smoking, and alcohol consumption	5
Petridou E	2002	Greece	hospital-based case–control study	N/A	FFQ 110 items	106/212	Meats and meat products	high vs low	energy intake, tobacco smoking, and alcohol consumption	5
Garrote LF	2001	Cuba	hospital-based case–control study	1996–1999	dietary questionnaire	200/400	Total meat, processed meat	Servings/week 3-5 vs <3 >6 vs <3	gender, age, area of residence, education, smoking and drinking habits	6
Zheng T	1993	China	hospital-based case–control study	1989	FFQ 63 items	404/808	Total meat	Servings/month 1-2 vs <1 >3 vs <1	tobacco smoking, alcohol drinking, inadequate dentition, years of education, Quetelet Index, sex and age	4
Zheng W	1992	China	population-based case-control study	1988–1990	FFQ 42 items	204/618	Salted meat	daily/weekly vs seldom	smoking and education.	5

BMI =  body mass index; FFQ =  Food Frequency Questionnaire.

### Total meat intake and the risk of oral cavity and oropharynx cancer

**Figure 2:**
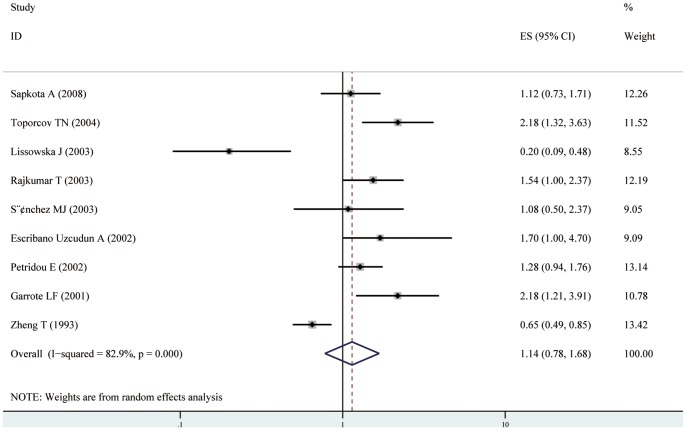
Forest plot: estimates (95% CIs) of total meat consumption and risk of oral cavity and oropharynx cancer. Squares indicated study-specific risk estimates (size of square reflects the study-statistical weight, i.e. inverse of variance); horizontal lines indicate 95% confidence intervals; diamond indicates summary relative risk estimate with its corresponding 95% confidence interval.

Nine case–control studies of total meat distinction were included in the meta-analysis [8], [9], [10], [11], [21], [23], [24], [25], [26]. We found that the high consumption of total meat was not significantly associated with the risk of oral cavity and oropharynx cancer (RR = 1.14, 95% CI[0.78–1.68]) (shown in Table 2, Figure 2). Statistically significant heterogeneity was detected (I^2^ = 82.9%, Q = 46.87, P<0.001). There was no indication of a publication bias, either from Egger ’s test (P = 0.780) or from Begg ’ s test (P = 0.835 )(shown in Figure 4 A). In subgroup analyses, when stratified the various studies by study location, no significant association was noted among studies conducted in Europe (RR = 0.93, 95%CI [0.55, 1.59]), and Asia (RR = 0.98, 95%CI [0.42, 2.29]), however, high consumption of total meat was significantly associated with a 118% increased risk of oral cavity and oropharynx cancer in South America (RR = 2.18, 95%CI [1.49, 3.20]). When we examined whether the associations were affected by adjustment for alcohol, BMI, education, and fruit and/or vegetable intake, the associations were not affected by these factors(shown in Table 2). Further, it was observed that studies with higher control for potential confounders (n≥7) as well as studies with lower control (n≤6) presented no significant association (RR = 1.02, 95% CI[0.59, 1.74] and RR = 1.21, 95% CI[0.70, 2.09], respectively). Sensitivity analysis indicated that no significant variation in combined RR by excluding any of the study, confirming the stability of present results. Meta-regression analysis was performed to investigate the possible sources of between-study heterogeneity. Geographic area, sex, age, study quality, publication year, control for confounding factors(alcohol, BMI, education, and fruit and/or vegetable intake) which may be potential sources of heterogeneity, were tested by a meta-regression method. We found that study quality( P = 0.029) had statistical significance in a multivariate model.

**Figure 3:**
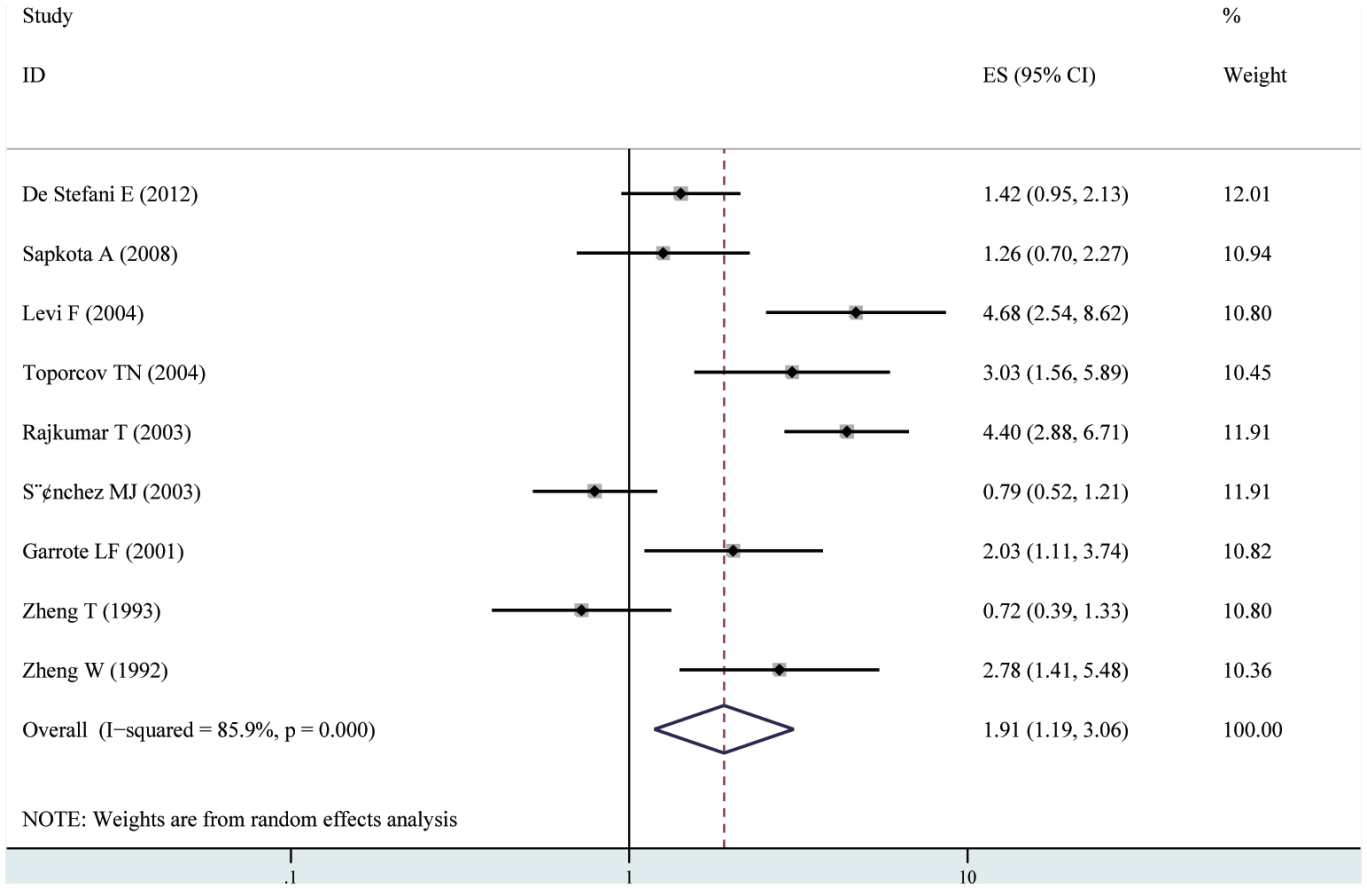
Forest plot: estimates (95% CIs) of processed meat consumption and risk of oral cavity and oropharynx cancer. Squares indicated study-specific risk estimates (size of square reflects the study-statistical weight, i.e. inverse of variance); horizontal lines indicate 95% confidence intervals; diamond indicates summary relative risk estimate with its corresponding 95% confidence interval.

**Figure 4:**
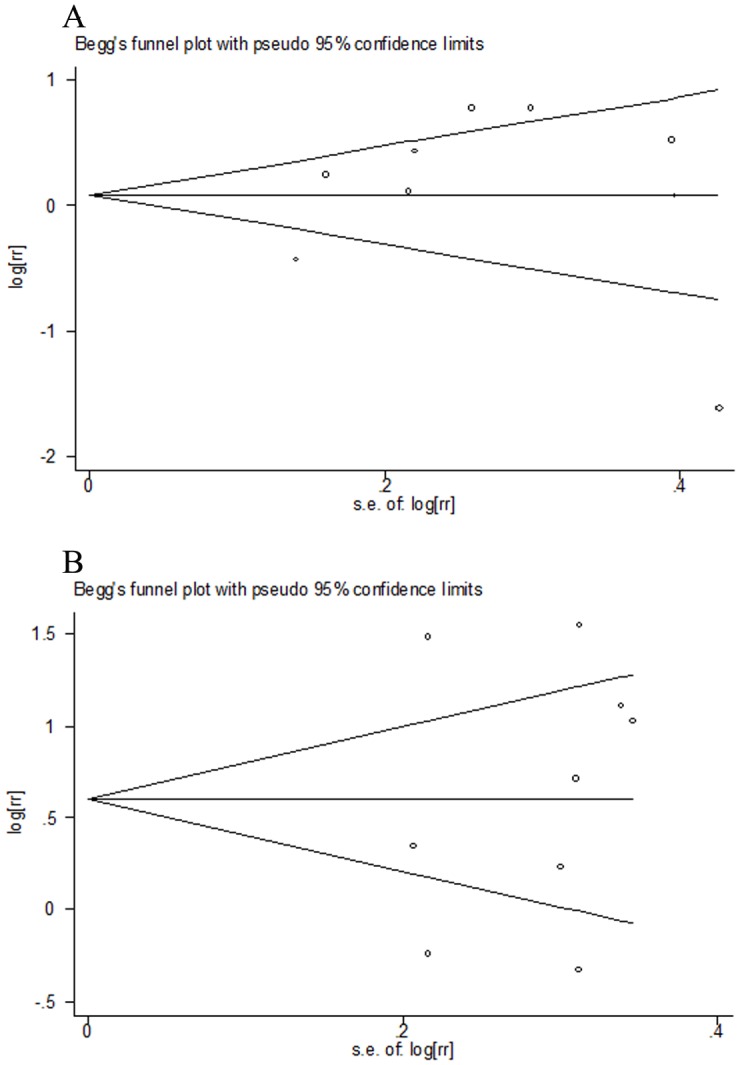
Begger's funnel plot of publication. A: Funnel plot for studies investigating total meat consumption and risk of oral cavity and oropharynx cancer; B: Funnel plot for studies investigating processed meat consumption and risk of oral cavity and oropharynx cancer.

**Table 2:** Summary relative risks of the association between meat consumption and risk of oral cavity and oropharynx cancer.

	No. of studies	RR (95% CI)	P value for heterogeneity	I^2^ value (%)
Overall studies				
Total meat	9	1.14 (0.78–1.68)	<0.001	82.90
Processed meat	9	1.91 (1.19–3.06)	<0.001	85.90
Red meat	3	1.05 (0.66–1.66)	0.12	49.40
White meat	3	0.81(0.54–1.22)	0.09	59.40
Subgroup analyses for total meat				
Continent				
Europe	5	0.93 (0.55–1.59)	<0.001	77.80
South America	2	2.18 (1.49–3.20)	0.99	0.00
Asia	2	0.98 (0.42–2.29)	<0.001	90.90
Adjusted for confounders				
n≥7 confounders	3	1.02 (0.59–1.74)	<0.001	83.90
n≤6 confounders	6	1.21 (0.70–2.09)	<0.001	81.30
Major confounders adjusted				
BMI				
yes	1	1.12(0.73–1.71)	/	/
no	8	1.14(0.73–1.78)	<0.001	85.10
Alcohol				
yes	7	1.15(0.77–1.73)	<0.001	74.80
no	2	1.17(0.36–3.84)	<0.001	94.10
Education				
yes	5	1.18(0.75–1.88)	<0.001	80.50
no	4	1.04(0.48–2.28)	<0.001	87.30
Fruit and/or vegetable intake				
yes	1	1.12(0.73–1.71)	/	/
no	8	1.14(0.73–1.78)	<0.001	85.10
Subgroup analyses for processed meat				
Continent				
Europe	3	1.64(0.59–4.60)	<0.001	91.00
South America	3	1.93(1.25–3.00)	0.15	47.90
Asia	3	2.09(0.70–6.29)	<0.001	91.30
Adjusted for confounders				
n≥7 confounders	5	1.94(0.97–3.88)	<0.001	89.10
n≤6 confounders	4	1.86(0.92–3.74)	<0.001	82.60
Major confounders adjusted				
BMI				
yes	3	2.00(0.94–4.24)	<0.001	83.50
no	6	1.86(0.96–3.23)	<0.001	88.70
Alcohol				
yes	7	1.71(0.97–3.00)	<0.001	88.50
no	2	2.91(1.81–4.67)	0.86	0.00
Education				
yes	7	1.88(1.02–3.46)	<0.001	88.60
no	2	1.98(0.95–4.13)	0.06	72.70
Fruit and/or vegetable intake				
yes	3	2.00(0.94–4.24)	<0.001	83.50
no	6	1.86(0.96–3.23)	<0.001	88.70

BMI =  body mass index; CI = confidence interval; RR = relative risk.

### Processed meat intake and risk of oral cavity and oropharynx cancer

Nine case–control studies of processed meat distinction were included in the meta-analysis [9], [10], [11], [20], [22], [24], [25], [26], [27]. We found that the high consumption of processed meat was significantly associated with a 91% increased risk of oral cavity and oropharynx cancer (RR = 1.91, 95% CI [1.19–3.06]) (shown in Table 2, Figure 3). Statistically significant heterogeneity was detected (I^2^ = 85.9%, Q = 46.87, P<0.001). There was no indication of a publication bias, either from Egger ’s test ( P = 0.999) or from Begg ’ s test ( P = 0.297) (shown in Figure 4 B). In subgroup analyses, when stratified the various studies by study location, high consumption of total meat was significantly associated with a 93% increased risk of oral cavity and oropharynx cancer in South America (RR = 1.93, 95%CI [1.25, 3.00]), however, no significant association was noted among studies conducted in Europe (RR = 1.64, 95%CI [0.59, 4.60]), and Asia (RR = 2.09, 95%CI [0.70, 6.29]). When we examined if thorough adjustment of potential confounders could affect the combined RR, it was observed that studies with higher control for potential confounders (n≥7) as well as studies with lower control (n≤6) presented no significant association (RR = 1.94, 95% CI[0.97, 3.88] and RR = 1.86, 95% CI[0.92, 3.74], respectively). However, we found that the associations were significantly affected by adjustment for alcohol consumption and education (shown in Table 2). Sensitivity analysis indicated that no significant variation in combined RR by excluding any of the study, confirming the stability of present results. Meta-regression analysis was performed to investigate the possible sources of between-study heterogeneity. Geographic area, sex, age, study quality, publication year, control for confounding factors(alcohol, BMI, education, and fruit and/or vegetable intake) which may be potential sources of heterogeneity, were tested by a meta-regression method. However, meta-regression revealed that none of the above factors was responsible for the between-study heterogeneity.

### Red, white meat intake and the risk of oral cavity and oropharynx cancer

Three case–control studies of red meat distinction were included in the meta-analysis [9], [25], [26]. And two case–control studies [25], [26] and one cohort study [28] of white meat distinction were included in the meta-analysis. It was founded that neither red meat nor white meat was associated with an increased risk of oral cavity and oropharynx cancer(RR = 1.05, 95% CI[0.66, 1.66] and RR = 0.81, 95% CI[0.54, 1.22], respectively).

## Discussion

To our knowledge, it was the first meta-analysis evaluating the association between meat consumption and oral cavity and oropharynx cancer risk. Twelve case-control studies and one cohort study were included in the present analysis, involving 501,730 participants and 4,104 oral cavity and oropharynx cancer cases. In the present study, we found that the total meat consumption was not significantly associated with the risk of oral cavity and oropharynx cancer. When we investigated different types of meat and the risk of oral cavity and oropharynx cancer, we found that processed meat consumption was significantly associated with an increased risk of oral cavity and oropharynx cancer, however, no statistically significant association was observed between red meat or white meat intake and the risk of oral cavity and oropharynx cancer.

Processed meat (meat preserved by smoking, curing, salting, or by addition of chemical preservatives) has been found to be associated with several tumors, such as colorectal adenomas [3], esophageal cancer [4], bladder cancer [6], and renal cancer [7]. The finding of our meta-analysis was in line with these meta-analyses. Processed meat has been hypothesized to play a important role in carcinogenesis, owing to their high levels of saturated fat and heme iron content, and potent mutagens produced during high temperature cooking and meat processing or preservation, including N –nitroso compounds (NOCs) [29], polycyclic aromatic hydrocarbons(PAHs) [30], [31], and heterocyclic amines (HCAs) [32], [33]. NOCs intake may contribute to carcinogenesis at a variety of anatomic sites in animals [34]. PAHs and HCAs, which can form DNA adducts and induce genetic alterations characteristic of tumors, have been shown to be carcinogens in animal studies [35]. During subgroup analyses, after being stratified by study location, it demonstrated that processed meat consumption had a significant association with increased risk of oral cavity and oropharynx cancer in south Americans, however, it could not be validated in Asians or Europeans. So, the strength of the association varied greatly across ethnic groups. The possible reasons were considerable differences in genetic background, life styles and environmental factors. In addition, we should notice that there were only two included studies investigating meat intake and the risk of oral cavity and oropharynx cancer among Asians, and limited number of patients may limit us to detect stable effects in this population. Additional studies are warranted to further validate ethnic difference in the effect of meat intake on oral cavity and oropharynx cancer risk, especially in Asians.

Red meat is a source of heme iron, which is more bioavailable than non-heme iron. Heme iron is contributed to carcinogenesis in rodents by generating free radicals and inducing oxidative stress [36]. Heme iron has also been shown to induce endogenous formation of NOCs [37]. Previous meta-analyses have shown that red meat intake was associated with an increased risk of colorectal adenomas [3], esophageal cancer [4], lung cancer [5], bladder cancer [6], and renal cancer [7]. However, the result of the present meta-analysis showed that red meat consumption was not significantly associated with an increased risk of oral cavity and oropharynx cancer. We should notice that there were only three studies investigating the relationship between red meat intake and oral cavity and oropharynx cancer risk, which limited us to get a narrow confidence interval and detect stable effects. So, more studies focusing on red meat and oral cavity and oropharynx cancer risk are needed in the future.

The strength of the present meta-analysis lies in a large sample size (501,730 subjects and 4,104 oral cavity and oropharynx cancer cases) and no significant evidence of publication bias. All studies adjusted for smoking, and most studies adjusted for some potential confounders, including age, sex, and alcohol consumption. Furthermore, our findings were stable and robust in sensitivity analyses. However, several limitations to this meta-analysis should be noted. Firstly, as a meta-analysis of observational data, the possibility of recall and selection biases can’t be ruled out. Compared with case-control studies, cohort studies are less susceptible to bias due to their nature. However, the present meta-analysis included only one cohort study, so more prospective cohort studies are needed to confirm the association in the future. Secondly, we did not search for unpublished studies, so only published studies were included in our meta-analysis. Therefore, publication bias may have occurred although no publication bias was indicated from both visualization of the funnel plot and Egger’s test. Thirdly, none of the included studies adjusted for betel-quid chewing, which is one of the primary risk factors for oral cavity and oropharynx cancer. Lastly, due to different methods used to report meat intake among studies, we failed to carry out a dose-response analysis between meat intake and the risk of oral cavity and oropharynx cancer.

In conclusion, the present meta-analysis suggested that a high intake of processed meat was significantly associated with an increased risk of oral cavity and oropharynx cancer, while there was no significantly association between total meat, red meat or white meat and the risk of oral cavity and oropharynx cancer. More prospective cohort studies are warranted to confirm these associations.

## Supporting Information

Checklist S1 (DOC)
